# Dual Impact: Exercise and Fasting to Boost Brain Mitochondrial Health in AD

**DOI:** 10.1002/alz70855_105239

**Published:** 2025-12-24

**Authors:** Vivien Csikos, Brittany M Hauger, Taylor A. Strope, Colton R Lysaker, Edziu M Franczak, Colin S McCoin, John P Thyfault, Heather M Wilkins

**Affiliations:** ^1^ University of Kansas Medical Center, Kansas City, KS, USA; ^2^ University of Kansas Alzheimer's Disease Research Center, Kansas City, KS, USA; ^3^ University of Kansas Alzheimer's Disease Research Center, Fairway, KS, USA; ^4^ Department of Neurology, University of Kansas School of Medicine, Kansas City, KS, USA; ^5^ Department of Biochemistry and Molecular Biology, University of Kansas Medical Center, Kansas City, KS, USA

## Abstract

**Background:**

Alzheimer's disease (AD) is a progressive neurodegenerative disorder characterized by cognitive decline and memory loss. A key contributor to AD pathology is metabolic dysfunction, including impaired mitophagy, a specialized form of autophagy targeting damaged mitochondria. We hypothesize that exercise and fasting, known to enhance metabolic health, can synergistically stimulate autophagy and mitophagy, offering a potential therapeutic strategy for AD.

**Method:**

To investigate the cellular mechanisms underlying the potential benefits of exercise on mitochondrial health, we cultured primary mouse neurons and treated them with serum (1% for 24h) from sedentary or exercised mice. We measured mitochondrial biogenesis using a MitoTimer reporter and mitophagy using an EGFP‐mCherry COX8 reporter. We also measured p62 and LC3B protein levels following treatment with the autophagy inhibitor chloroquine (CQ). We also investigated the impact of exercise and fasting on autophagy in vivo using proteomics combined with CQ inhibition of autophagic flux in 5xFAD AD mice and their WT littermates (*n* = 48, 12‐15 weeks of age). The animals were divided into control (Sed, *n* = 6) and combined fasting with exercise (FEx, *n* = 6) groups. Post‐intervention, the animals received unilateral intrahippocampal CQ or PBS injections, and 4 hours post‐injection, the hippocampi were collected for analysis.

**Result:**

Exercise serum increased mitochondrial biogenesis and mitophagy in primary neurons. We observed increased autophagic flux in exercise serum treated neurons by increased LC3‐II and p62 content following CQ treatment. Proteomic analysis revealed distinct mitochondrial adaptations between WT and 5xFAD mice. While both strains showed upregulated metabolic pathways (TCA cycle and ETC) following an acute bout of FEx, 5xFAD mice exhibited a blunted response. Antioxidant defense adaptations to acute FEx were prominent in WT mice but absent in 5xFAD animals. Additionally, acute FEx robustly upregulated lysosomal proteins in WT mice but not in 5xFAD mice. Autophagy and mitophagy were differentially modulated by FEx, suggesting impaired adaptive responses in 5xFAD mice.

**Conclusion:**

These findings highlight the potential of exercise and fasting to enhance mitochondrial and lysosomal function, though their efficacy may be compromised in the context of AD pathology.

